# Urinary symptoms and prostate cancer—the misconception that may be preventing earlier presentation and better survival outcomes

**DOI:** 10.1186/s12916-022-02453-7

**Published:** 2022-08-04

**Authors:** Vincent J. Gnanapragasam, David Greenberg, Neil Burnet

**Affiliations:** 1 Department of Urology, Cambridge University Hospitals Trust, Cambridge, UK; 2 Division of Urology, Department of Surgery, University of Cambridge, Cambridge, CB2 0QQ UK; 3 Cambridge Urology Translational Research and Clinical Trials Office, Cambridge Biomedical Campus, Cambridge, UK; 4 (Retired) Public Health England, Public Health England, National Cancer Registration and Analysis Service (Eastern Office), England, UK; 5 The Christie NHS Foundation Trust, Manchester, UK

**Keywords:** Prostate cancer, Lower urinary tract symptoms, Early detection, Symptoms

## Abstract

**Background:**

Prostate cancer is an epidemic of the modern age, and despite efforts to improve awareness, it remains the case that mortality has hardly altered over the decades, driven largely by late presentation. There is a strong public perception that male urinary symptoms is one of the key indicators of prostate cancer, and this continues to be part of messaging from national guidelines and media health campaigns. This narrative, however, is not based on evidence and may be seriously hampering efforts to encourage early presentation.

**Discussion:**

Anatomically, prostate cancer most often arises in the peripheral zone, while urinary symptoms result from compression of the urethra by prostatic enlargement more centrally. Biopsy studies show that mean prostate volume is actually lower in men found to have (early) prostate cancer compared to those with benign biopsies. This inverse relationship between prostate size and the probability of cancer is so strong that PSA density (PSA corrected for prostate volume) is known to be significantly more accurate in predicting a positive biopsy than PSA alone. Thus, this disconnect between scientific evidence and the current perception is very striking. There is also evidence that using symptoms for investigating possible cancer may lead to higher proportions of men presenting with locally advanced or metastatic disease compared to PSA testing or screening programmes. Concerns about overwhelming health care services if men are encouraged to get tested without symptoms may also be overstated, with recent newer approaches to reduce over-investigation and treatment. In this article, we explore the link between urinary symptoms and prostate cancer and propose that public and professional messaging needs to change.

**Conclusion:**

If rates of earlier diagnosis are to improve, we call for strong clear messaging that prostate cancer is a silent disease especially in the curable stages and men should come forward for testing regardless of whether or not they have symptoms. This should be done in parallel with other ongoing efforts to raise awareness including targeting men at highest risk due to racial ancestry or family history. While the current resurgence in interest and debate about prostate cancer screening is timely, change of this message by guideline bodies, charities and the media can be a first simple step to improving earlier presentation and hence cures rates.

## Key message

- Waiting for troublesome lower urinary tract symptoms as a trigger to see a GP about prostate cancer may potentially delay earlier diagnosis and management.
- There is no evidence of a causal link between prostate cancer and either prostate size or troublesome male urinary symptoms. In fact, most evidence points to an inverse correlation.
- Modern image-based diagnostics and risk-adapted management strategies have reduced the risks of over-investigation and over-treatment which previously deterred greater promotion of PSA testing in men with no symptoms.
- It is now timely to re-brand early, curable prostate cancer as primarily an asymptomatic disease to encourage more men to come forward and get tested earlier.

## Background

There is a strong public perception that male urinary symptoms are a sign of prostate cancer. Yet, this notion is not borne out by the scientific evidence. Research over the last 30 years has consistently found no direct relationship between symptoms and prostate cancer. Despite this, the link between urinary symptoms and prostate cancer continues to be reinforced by national guidelines and media health promotion messaging. In this article, we review the scientific evidence base and how this perceived causative association may actually deter men from coming forward for early testing and detection of treatable cancer.

### Prostate cancer—still a disease of late presentation and unchanged mortality rates

Prostate cancer is without a doubt a disease epidemic of the modern age. Although first described histologically only in 1853, as a single case report, today, it is actually the most common male cancer in the western world today and a major cause of morbidity and mortality [1, 2]. The annual incidence is over 52,000 cases, and there are almost 12,000 deaths each year, 7% of all cancer deaths (https://www.cancerresearchuk.org/about-cancer/prostate-cancer/symptoms). Although considered typically a disease of older age, more than a quarter of cases occur before current retirement age. Despite significant recent advances in treatment, over the last decade, the proportion of men dying of prostate cancer has hardly altered in the UK and many countries, driven largely by late presentation of the disease. In England, for example, nearly half of all prostate cancers present as stage 3 or 4 (https://www.npca.org.uk/content/uploads/2021/01/NPCA-Annual-Report-2020_-Infographic-140121.pdf/). On the face of it, it would seem that screening should also be introduced to reduce prostate cancer mortality, and this has been robustly debated over many years [3]. The converse argument is that this may result in many men being unnecessarily investigated and treated for likely indolent disease. As a result, outside of North America, few PSA-based testing programs have been introduced internationally. Recently, there has been a resurgence of interest in screening methods, particularly with the advent of prostate MRI which has shown high negative predictive values, thus reducing the numbers of men who proceed to biopsy [4]. Nevertheless, it remains to be seen if proposed new approaches (e.g. targeted genetic testing, MR imaging) will fulfil the Wilson and Jungner criteria for a screening programme [5–8]. In the meantime, it is important to consider how men currently seek advice about suspected prostate cancer, the recommendations in terms of testing and whether these remain fit for purpose.

### Male urinary symptoms and prostate cancer—causality, association or no link at all?

For decades, the recommendation has been to use symptoms to look for possible cancer, in particular lower urinary tract symptoms (LUTS). Yet, the origins and evidence for this linkage are hard to uncover. ‘Urinary symptoms as a sign of prostate cancer’ is often part of the thrust of messages from the media and is included in information from national guidelines and charities as a means to encourage men to get tested (https://www.nhs.uk/conditions/prostate-cancer/symptoms, https://www.cancercenter.com/cancer-types/prostate-cancer/symptoms, https://www.verywellhealth.com/symptoms-of-prostate-cancer-2782274). Although many information and official sites do mention that most prostate cancers may not cause symptoms, they do still very prominently list a number of urinary symptoms to look out (https://www.nhs.uk/conditions/prostate-cancer/symptoms, https://www.cancercenter.com/cancer-types/prostate-cancer/symptoms, https://www.verywellhealth.com/symptoms-of-prostate-cancer-2782274). This is based on the notion that prostate cancer may manifest with symptoms such as slow urinary flow, frequency or nocturia which should trigger a visit to the GP for testing to exclude cancer. Indeed, the UK National Institute for Health and Care Excellence (NICE) makes a distinction between patients with and without symptoms in how to manage suspected prostate cancer, for example recently reviewing the evidence base for PSA thresholds, but only in the ‘symptomatic’ population [9]. Much of the basis for this distinction has come from studies in primary care-based referral practices of symptomatic men. The widely used QCancer® tool was developed using data from over 2 million men presenting to GPs. For prostate cancer, urinary retention, frequency, urgency and impotence were considered predictors of a diagnosis. However, PSA is not part of the model and there is no detail on the stage of cancers found through this route [10]. Staying with primary care, in an analysis piece from 2018, Just et al. debated the use of PSA in men presenting with urinary symptoms and pointed out the lack of evidence of its utility in cancer detection and that in fact current practice may lead to over-diagnosis of indolent cancers [11]. More recently. Koo et al. [12], as part of a wider study of primary care-detected malignancy, analysed a cohort of 1135 men who saw their GP for LUTS and were subsequently diagnosed with prostate cancer. In this sub-group, they reported that in diagnosed men nearly 20% already had stage IV, i.e. metastatic disease. This rate is much higher than in men investigated through a raised PSA route and also higher than the national overall rate of metastatic disease in men diagnosed through any route (Table 1) (https://www.npca.org.uk/content/uploads/2021/01/NPCA-Annual-Report-2020_-Infographic-140121.pdf/). There are no direct comparison studies of detection rates and stage differences in PSA-detected versus symptom-detected cancers. So, is there evidence of a causal link between urinary symptoms and prostate cancer and to justify managing men differently?

**Table 1:** Comparison of reported proportions of men detected with incurable metastatic/stage VI prostate cancer through different detection strategies. These studies were done in different populations as indicted and based on different investigation indications

Type of cohort studied	Percent of cancers found presenting with Stage IV/Metastasis at diagnosis	Cited publication
Symptomatic men seen in primary care and referred	19%	Koo et al. 2020 [12]
Population based reporting National Prostate Cancer Audit 2019 England and Wales	13%	NPCA report 2019 (https://www.npca.org.uk/content/uploads/2021/01/NPCA-Annual-Report-2020_-Infographic-140121.pdf/)
Screening study, men PSA ≥ 3 ng/ml (Gothenburg screening trial)	2.4%	Hugosson et al. [13]
Screening study, men PSA ≥ 3 ng/ml (ERSPC screening trial)	2.5%	Schröder et al. [14]

#### Prostate cancer and gland size

Bladder outlet obstruction (most often due to benign prostate enlargement) and its symptomatic manifestations, difficulty with flow, hesitancy, nocturia and poor stream, are the most common symptoms taking a man to his GP and leading to a PSA test. The relationship between prostate size and cancer has been extensively explored since the earliest days of routine PSA and biopsies. Karakieweiz et al. were amongst the first to report that mean prostate volume was lower in men found to have prostate cancer compared to those with benign biopsies [15]. Since then, other studies have reported identical findings. In a meta-analysis compiled by Moolupuri et al., 28 out of 30 studies showed a clear inverse relationship between prostate size and the chance of finding prostate cancer at biopsy [16]. The remaining 2 studies were equivocal and none showed a positive correlation. This relationship is so strong that the PSA density (PSA corrected for prostate volume) is now well known to be significantly more accurate than PSA in predicting a positive biopsy and is used in everyday clinical practice [17]. In an elegant computer simulation, Lorenzo et al. postulated that an enlarged prostate could in fact cause mechanical suppression of tumour growth, which may explain the inverse size relationship with cancer detection [18]. One caveat is that many of these studies were done before pre-biopsy MRI imaging to target biopsies (as in modern pathways) and tumours may have been harder to find in larger prostates. However, a recent study from our own unit has identified that size-cancer relationship remained the same (inverse) in men investigated by MRI-guided biopsies [19].

#### Lower urinary tract symptoms (LUTS) and prostate cancer

Overall size alone of course does not explain all lower urinary tract symptoms. Anatomically, early stage cancer should not be expected to cause urinary symptoms. The most common site for malignancy (70%) is in the peripheral zone, while urinary symptoms as a result of (benign) prostatic enlargement occur because of growth of the transitional and central zones. The origins of a causal linkage between LUTS and cancer are hard to uncover but likely relate to the pre-PSA era before formal studies on this topic [20]. One of the only large epidemiological studies to report an association was the HUNT 2 study (conducted between 1995 and 2007 in Norway). But even this study found a paradoxical link with localised, but not advanced or fatal, prostate cancer [21]. The authors concluded that urinary symptoms were not caused by prostate cancer and that screening for early cancers on the basis of urinary symptoms was not justified. There is more recent level 1 data from randomised controlled screening trials about LUTS and prostate cancer. Nearly 10 years ago, the Gothenburg screening trial investigated the incidence of urinary symptoms and detection of cancer in men with a raised PSA (using a threshold of ≥ 3 ng/ml) [22]. Not only did they not find an association but they observed an inverse relationship between symptoms and the chance of a positive biopsy. More recently, the UK PROTECT trial also looked in detail at the LUTS scores that men reported: for every domain of urinary score there was either no association or a negative association with more severe symptoms and prostate cancer [23]. The authors concluded that a lack of urinary symptoms may in fact be an indicator of a higher likelihood of cancer. These and many other similar studies provide quite convincing evidence that LUTS and prostate cancer are not positively linked, may in fact be inversely associated, and when present are more likely to be (at most) co-incidental [24, 25].

### An urgent need to change the message

So where does this leave the notion of prostate cancer as a ‘symptomatic disease’? The idea that LUTS and cancer are causally associated is so strong that in a study looking at population-based awareness, 86% of the public associated prostate cancer with symptoms, but only 1% were aware that it could be asymptomatic [26]. Given the data above, this disconnect between scientific evidence and public perception is extremely striking. We believe that the time has come to urgently revaluate the messaging on urinary symptoms and recognise that the information currently being passed to the public is factually wrong. Any link between urinary symptoms and prostate cancer should be in our view removed as it risks men having a false sense of security if they do not have any urinary systems. Instead, there is a strong basis to emphasise that prostate cancer is silent or asymptomatic particularly in the curable stages. Waiting or looking out for urinary symptoms may potentially give a false sense of reassurance that all is well and delay presentation when the disease is treatable. Nevertheless, we are not advocating that patients with urinary symptoms be discouraged from presenting for investigation but rather that it should be recognised that male urinary symptoms cannot be used as a cardinal indicator of prostate cancer. Clearly, the downside of this change in message approach is that more men may approach GPs for a PSA test with the accompanying fears of over-investigation and treatment. However, there are a number of strategies that can already or will in future be able to reducing both concerns. At the primary care stage novel algorithms to risk assess patients for referral have been recently published using PSA combined with demographic and co-morbidity factors in a stepped approach to help decide who to refer [27]. With the use of MRI in diagnostic pathways to rule out indolent disease or negative findings, the risks of proceeding to an unnecessary biopsy after referral have also been largely mitigated (up to 40% reduction) [4]. The European Association of Urology for example has recently published recommendations for a risk-adapted early detection strategy using age-based PSA, testing schedules as well as MRI to rationalise who needs investigation (and who does not) [28]. In addition, after diagnosis, the rising use of active surveillance to manage early prostate cancer means that over-treatment is already much less of an issue. As an example, the National Prostate Cancer Audit in 2019 estimated that amongst men with favourable prognosis disease only 5% currently receive active treatment, a far cry from the days of previous screening studies (https://www.npca.org.uk/content/uploads/2021/01/NPCA-Annual-Report-2020_-Infographic-140121.pdf/). Conversely, proponents of keeping the status quo may argue that that any testing is better than none and using LUTS to get men to a primary care doctor may be a ‘win’, regardless of whether or not there is an association. However, this could actually be considered to itself be a ‘screening strategy’ and one that has been shown to not be an effective way to detect cancer in the pre-PSA era [21]. As can be seen by the data in Table 1, it is also the strategy which is most likely to result in a higher proportion of men detected with later stage disease. Prospective research is also needed to explore the current contrasting findings of PSA-detected versus symptom-detected cancers and its impact on stage at diagnosis and the longitudinal survival effect of waiting till symptoms lead to presentation.

## Conclusion

We therefore call on guideline bodies, charities and the media to take urgent action to review the current public messaging and referral recommendations. Paramount is to abolish the public messaging suggesting that prostate cancer directly causes urinary symptoms. To maintain this fallacy is misleading. Efforts should instead be made to raise awareness that prostate cancer does not manifest with urinary symptoms. To be clear, we are not here advocating for an immediate screening programme nor are we asking to change already existing pathways (e.g. serendipitous investigation of men seeing primary care doctors for urinary symptoms). We recognise that the latter remains an important route for men to be detected and in particular for men who may not access health information resources or in hard to reach socio-demographics groups. For many of these men, the only reason to see a doctor is if they are having manifest bothersome symptoms. However, if men were aware that just because they had no symptoms did not mean they may not have cancer, more men might take up offers for tests. This could mean more tumours identified at an earlier stage and reduce the numbers of men experiencing late presentation with incurable disease. Eventually, we hope that an intelligent tiered screening programme will be possible, but until then, a simple change in message to correct years of misinformation would be a strong starting point.

## Data Availability

There is no new data or materials used in this article. Data sharing is not applicable to this article as no datasets were generated or analysed during the current study.
